# Wilson Disease in Children in the Eastern Region of Morocco: Analysis of 24 Cases

**DOI:** 10.7759/cureus.60023

**Published:** 2024-05-10

**Authors:** Maria Rkain, Massilia Bouhmidi, Amal Hamamı, Aziza Elouali, Siham Chariba, Imane Kamaoui, Imane Skiker, Abdeladim Babakhouya

**Affiliations:** 1 Department of Pediatrics, University Hospital Mohammed VI, Faculty of Medicine and Pharmacy, Mohammed First University, Oujda, MAR; 2 Department of Ophthalmology, University Hospital Mohammed VI, Faculty of Medicine and Pharmacy, Mohammed First University, Oujda, MAR; 3 Department of Radiology, University Hospital Mohammed VI, Faculty of Medicine and Pharmacy, Mohammed First University, Oujda, MAR

**Keywords:** screening., d-penicillamine, central nervous system, kayser-fleischer ring, hepatopathy, atp7b mutations, copper, child, wilson

## Abstract

Wilson's disease (WD), or "hepato-lenticular degeneration," is a rare genetic disorder of autosomal recessive inheritance causing toxic tissue accumulation of copper, mainly in the liver, brain, and cornea. Its phenotypic and genotypic heterogeneity characterizes it. This study aimed to clarify the clinical features and spectrum of Wilson's disease in children from the eastern region of Morocco and to study the evolutionary profile and survival in this population while discussing and highlighting the various diagnostic and therapeutic difficulties encountered in the management of WD in our context. This retrospective study encompassed 24 children diagnosed with Wilson's disease, selected from the gastroenterology-hepatology and pediatric nutrition units at Mohamed VI University Hospital in Oujda, Morocco, over a span of nine years, from January 2015 to November 2023. Our series results show 14 boys and 10 girls; the median age of discovery was 11 years, with extremes ranging from 18 months to 15 years. The consanguinity was found in 13 patients. Clinically, the edemato-ascitic syndrome was noted in 14 patients with an alteration of the general state; icterus was found in 13 patients; signs of portal hypertension were present in six patients; and neurological signs in seven cases. Skin manifestations occurred in three cases, and arthralgia in three cases. Six children were diagnosed on the occasion of a family screening. Biologically, hepatic cytolysis was found in 20 patients, with signs of hepatocellular failure in 15 cases. Hemolytic anemia was present in nine patients. Ceruloplasminemia was decreased in 21 patients and cupremia in 19 patients. Cupruria was increased in 22 cases. The Kayser-Fleicher ring was found in 10 cases. Abdominal ultrasound showed ascites in 16 patients, hepatomegaly in 1, splenomegaly in two cases, hepatosplenomegaly in five cases, and cirrhosis in two. MRI showed signal abnormalities in 11 patients. Therapeutically, D-penicillamine was initially introduced in 18 patients and zinc acetate in 6 patients. The evolution was favorable for 15 patients still followed up in the department. Three patients died of hepatocellular failure, and two died of hepatic encephalopathy. Four patients were lost to follow-up.

## Introduction

Wilson's disease (WD) is a rare autosomal recessive genetic disorder affecting copper metabolism. It is a copper toxicosis characterized by tissue accumulation of free copper, mainly in the liver, brain, and pericornea [1,2]. It results from mutations in the ATP7B gene on chromosome 13. This ATP7B protein transports copper in the hepatocyte. In France, 906 cases of WD were identified in 2013, representing a clinical prevalence of 15.4 cases per million inhabitants [2]. In Morocco, it remains underestimated due to the difficulty of diagnosis. The age of onset of the disease is often between 10 and 20 years, but it can occur at extreme ages [3]. Diagnosis is based on a number of factors: clinical, biological (cardiac work-up), radiological (MRI), and even histological [4]. This rare genetic disease has a good prognosis if treatment is initiated early and continued for life. In the absence of any treatment, spontaneous evolution is usually fatal. Currently, several therapeutic options are available to stabilize the disease and avoid the damage associated with copper accumulation, subject to good compliance [2-4].

Our work aimed to clarify the clinical features and spectrum of Wilson's disease in children from the eastern region of Morocco and to study the evolutionary profile and survival in this population while discussing and highlighting the various diagnostic and therapeutic difficulties encountered in the management of WD in our context.

## Materials and methods

We conducted a retrospective study of 24 children followed in the Pediatric Gastroenterology-Hepatology and Nutrition Unit in the Pediatrics Department of the Mohamed VI Hospital and University Center in Oujda over a nine-year period from January 2015 to November 2023.

The inclusion criteria used in our series were children under 16 years of age with liver damage and/or neurological impairment, disturbed copper balance, and/or the presence of a Kayser-Fleischer ring. We have excluded children aged over 16 who had a normal copper balance. Data were collected from the patient's medical records. An Excel file (Microsoft® Corp., Redmond, WA) was created to collect anamnestic, clinical, biological, radiological, and therapeutic data.

We analyzed clinical, biological (hepatic and cupric), and evolutionary characteristics. Family screening was carried out by clinical examination and analysis of the siblings' copper levels.

## Results

The average number of cases in our series was three per year. The average age of onset of Wilson's disease was 11 years, with extremes of 18 months and 15 years. The age group most affected was 8 to 12 years old. Among all patients, there was a predominance of males, with 14 boys out of 24 (58%) and 10 girls (42%), giving a sex ratio (M/F) of 1.4.

Consanguinity was found in 13 patients (54%). Nine of them had first-degree consanguinity; the other four had second-degree consanguinity. The case histories of our patients included eight cases of death in siblings with similar symptoms, one child who had failed at school, four cases of recurrent epistaxis, and one child with chronic headaches. All our patients had a progressive onset of clinical manifestations. However, the delay between the appearance of the first clinical signs and the diagnostic consultation varied from 6 days to 12 months, with an average of two months. The main reason for consultation was the edemato-ascitic syndrome, seen in 14 children (58%), with edema of the lower limbs and ascites, and an altered general state, with asthenia, anorexia, and weight loss, seen in 18 patients (75%). Jaundice was present in 13 patients, i.e., half the cases, and digestive signs (abdominal pain, nausea, vomiting, and diarrhea) in seven patients. Seven out of twenty-four children had hepatomegaly; collateral venous circulation was present in seven patients; and splenomegaly was present in six children as part of portal hypertension syndrome (PHS).

Neuropsychiatric signs were present in seven patients aged between 8 and 15 years. The average time to onset of clinical signs in these seven cases was two months, with extremes of 13 days and 7 months. The neurological examination revealed extrapyramidal signs in four cases (slowness of gestures and ideation, frozen face), a sardonic smile in one case, a behavioral disorder in two cases, and micrography in three cases, one of which was associated with one of the two patients with a behavioral disorder.

Three patients presented with arthralgia and/or limited joint mobility, and five patients were asymptomatic at the time of screening among the siblings of the patients in our series. Regarding the dermatological manifestations of WD, cutaneous hyperpigmentation was noted in two patients in our series and one case of cutaneous xerosis. Clinical signs are shown in Table 1.

**Table 1:** Clinical findings in our series.

Clinical signs	Number	Percentage (%)
Icterus	13	54
Edemato-ascitic syndrome	14	58
Hepatomegaly	7	29
Splenomegaly	6	24
Collateral venous circulation	7	29
Extrapyramidal signs	4	17
Sardonic face	1	4
Behavioral disorder	2	8
Micrography	3	12
Arthralgia	3	12

**Figure 1:**
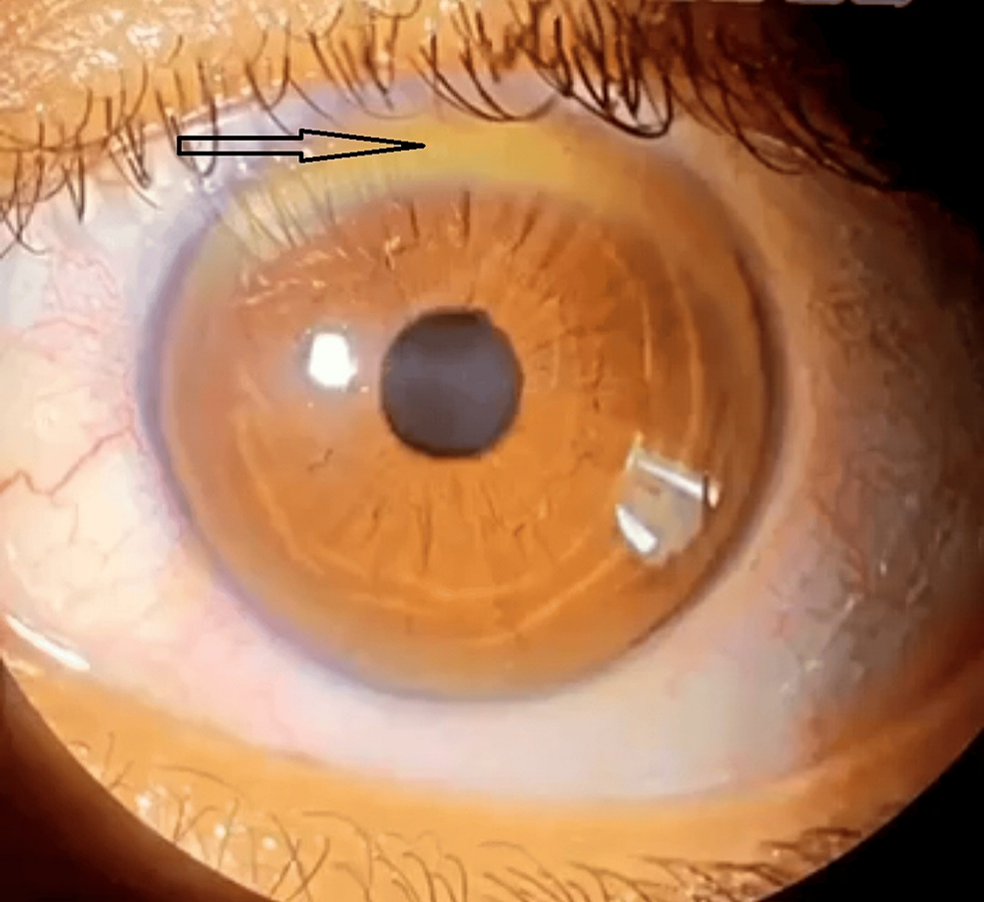
Slit lamp image of Kayser Flesicher ring of one of our patients.

Slit-lamp ophthalmological examination revealed the presence of the Kayser-Fleisher ring (KFR) in 10 patients, i.e., 42% of cases (Figure 1), nine of whom were over eight years old and one six-year-old with incipient KFR. The mean time for disease progression was five weeks.

Biologically, transaminases were normal in four patients (17%), moderately elevated in 11 (46%), and very elevated in nine children (37%). Prothrombin level (PT) was below 40% in 13 patients (54% of cases), between 40% and 70% in two children, and normal in nine patients, six of whom had undergone family screening. Factor V was performed in 11 patients and collapsed in 8. Hypoalbuminemia was noted in 16 patients, i.e., 67% of cases. Protein electrophoresis (PEP), performed in seven patients, showed hypoalbuminemia in all cases, associated with polyclonal hypergamma-globulinemia characterized by a beta-gamma-globulinemia block compatible with hepatocellular failure in six cases.

Hematologically, hemolytic anemia was found in nine patients (37%), thrombocytopenia in 11 (46%), and moderate leukopenia with WBC counts of between 1,500 and 4,500 elements/mm3 in four patients.

Ceruleoplasminemia was reduced in 21 patients (88%), normal in one child (4%), and not performed in two cases. Cupremia was reduced in 19 patients (79%), increased in one, and not performed in four. Cupruria was performed in 22 patients and was found to be increased in all children. Relative exchangeable copper (REC) was measured in nine patients and found to be elevated (Table 2).

**Table 2:** Our patients' copper balance sheet. Low, standard, and high rates of each copper balance element.

Copper balance (patient numbers)	Low rate (patient numbers)	Standard rate (patient numbers)	High rate (patient numbers)
Ceruleoplasminemia (22)	21	1	0
Cupremia (20)	19	0	1
Urinary copper excretion (22)	0	0	22
REC (9)	0	0	9

Abdominal ultrasonography showed ascites in 16 patients (67%) associated with cirrhosis of the liver in two cases, hepatosplenomegaly in five cases, hepatomegaly alone in one case, and splenomegaly alone in two cases. Hepatosplenomegaly without ascites was noted in two cases and isolated hepatomegaly in two children. The other patients were diagnosed by family screening and had normal ultrasounds.

**Figure 2:**
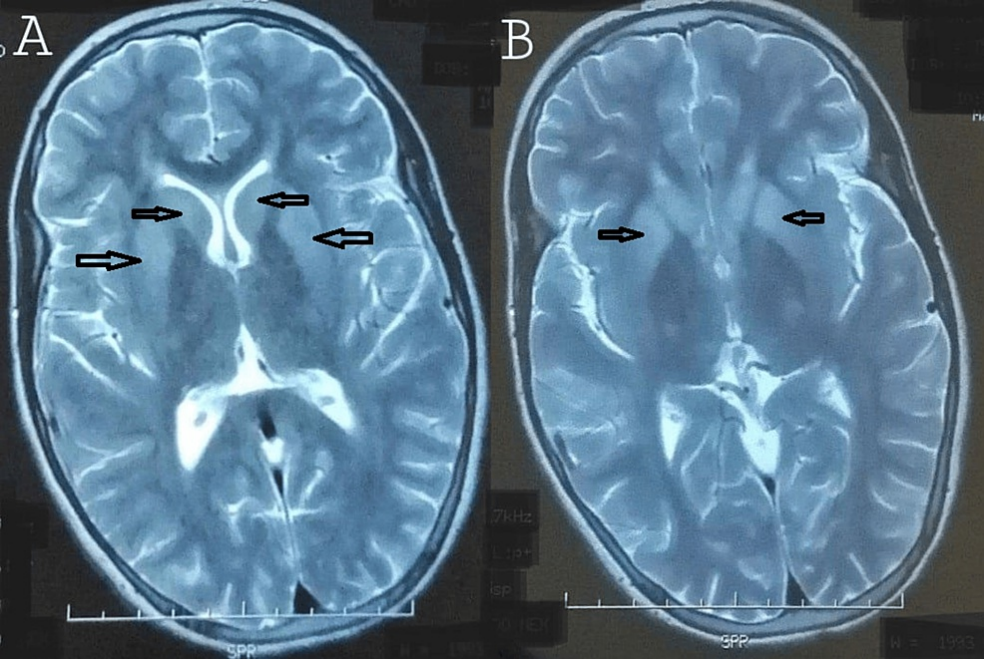
Cerebral MRI axial sections in T2 sequence. (A) Bilateral and symmetrical basal ganglia T2 hyperintensity; (B) hypersignal of the basal ganglia.

Digestive fibroscopy was performed on three patients. It showed stage I esophageal varices without red signs in one patient and moderate antritis in another. In the remaining case, eso-gastroduodenal fibroscopy was normal. A cerebral MRI was carried out in all our patients and revealed bilateral and symmetrical T1 hyper-signal of the pallidum in four cases, bilateral and symmetrical involvement of the basal ganglia in four cases (T2 hypersignal; Figure 2), bilateral and symmetrical involvement of the lenticular and mesencephalic nuclei in one case, a giant Panda head appearance in one case and an appearance consistent with leukodystrophy in another. In the remaining cases, MRI scans showed no abnormalities. A liver biopsy was performed on only one patient in our series, showing chronic liver disease. It was not performed in the other patients, given the severe liver damage and very low prothrombin levels.

In terms of treatment, all the children and their parents benefited from a dietary consultation with the introduction of a low-copper diet. Eighteen of our patients were treated with D-penicillamine (TROLOVOL® /CUPRIPEN®), with doses gradually increased to two to three times daily on an empty stomach, reaching the full dose by the fourth week. Of these 18 patients, three were switched to zinc acetate due to severe toxidermia with hematological damage in one patient and the unavailability of D-penicillamine on the Moroccan pharmacological market in two patients. The last six patients were treated immediately with zinc acetate (WILZIN®), with the dosage depending on the age and weight of each patient. None of the patients received vitamin B6. Liver transplantation was not indicated in any patient in our series.

As adjuvant therapy, 14 patients with edemato-ascitic syndrome were started on diuretics (spironolactone, furosemide) at a dose of 2-4 mg/kg/d with diuresis monitoring, and 15 patients were started on vitamin K (10 mg/d), in view of clinical and biological cholestasis and low PT, in addition to transfusions of fresh frozen plasma (nine cases for hepatocellular insufficiency), platelet and red blood cells (one case with poorly tolerated anemia and severe thrombocytopenia), as well as albumin infusions (seven children).

Treatment-related side effects were noted in 11 patients, i.e., half the cases. Hematological side effects included 10 cases of thrombocytopenia and 6 cases of leukopenia. On the neurological front, two cases of neurological aggravation, such as agitation, were observed in connection with hepatic encephalopathy and generalized convulsive seizures (without post-critical deficit) one week after the initiation of D-penicillamine. On the gastrointestinal front, only one patient suffered mild hematemesis with melena due to an error in the administration of zinc acetate [75 mg divided into two doses (50 mg and 25 mg) instead of 3], necessitating transfusion of the patient with packed red blood cells and the administration of a PPI (2 mg/kg/d). Side effects such as nausea, vomiting, and disgust were observed in five patients. In addition, a case of severe cutaneous toxidermia (pruritic maculopapular lesions and purpuric spots) associated with hematological damage two days after the start of treatment, necessitating a change in treatment, and a case of stretch marks after administration of D-penicillamine were noted.

After an average follow-up of four and a half years, with extremes ranging from two months to nine years, the evolution was favorable, with good efficacy and clinico-biological tolerance, including the disappearance of symptoms, normal growth and schooling, and normalization of the biological balance, in 15 patients who are still being followed in the Pediatric Department. On the other hand, there were five deaths, three of which were the result of decompensated cirrhosis in patients who had consulted us late for edematous-ascitic syndrome, jaundice, and hepatocellular insufficiency, and two of which were the result of hepatic encephalopathy. In addition, the evolution of four patients could not be assessed because they had been lost to follow-up.

In our series, a family investigation was systematically carried out in all patients, with a copper work-up in the siblings. This investigation led to the diagnosis of five cases included in the series.

## Discussion

The genetic prevalence of WD in France is estimated at 30/1,000,00 [5]. In France, 906 cases of WD were identified in 2013, representing a clinical prevalence of 15.4 cases per million inhabitants [2]. Consanguinity considerably increases the incidence of WD [4]. Given the frequency of consanguineous marriages, it is thought to be fairly common in Maghreb countries. Its exact incidence in these countries is unknown. In Morocco, it remains underestimated due to the difficulty of diagnosis. The age of onset of the disease is often between 10 and 20 years, but it can occur at the extremes of life [3]. This disease is characterized by a diversity of clinical manifestations, dominated by hepatic, neurological, and ocular symptoms, which may be isolated, associated, or follow one another [2]. In children, the first symptoms are usually hepatic, whereas in adults, the symptoms are mainly neuropsychiatric. The disease may also manifest itself through renal, hematological, endocrine, or cardiovascular manifestations [6]. Some authors report that forms with a hepatic presentation have a risk of death five times higher than forms with a neurological presentation [7]. In our study, the first manifestations were hepatic in 22 patients (92%) and neurological in 2 patients (8%). Hepatic involvement is often isolated in children, discovered between the ages of 5 and 13. Several clinical pictures can be revealed, ranging from a simple increase in transaminases to fulminant hepatitis [8]. In our series, the majority of our patients presented immediately with cirrhosis, suggesting a delay in diagnosis probably due to three factors: most often, a delay in consultation, but sometimes also due to the clinical heterogeneity that makes the diagnostic hypothesis difficult, as well as a rapidly severe evolution, since the average time to progression found in Moroccan children with WD in the eastern region was two months.

Admittedly, the first symptoms are mainly hepatic in children, but in our series, we reported one child who presented purely with neuropsychiatric symptoms with no other associated damage, notably hepatic. Although neurological involvement is exceptional before the age of 12, we have described three children under the age of 12 (8 and 10 years old) whose neurological symptoms occurred after liver involvement in two cases and concomitantly with liver damage in only one child.

Gradually and without treatment, the classic neurological picture of the disease develops a dystonic syndrome characterized by dystonic postures and choreoathetotic movements, an ataxic syndrome with postural and intention tremor and limb ataxia, and an extrapyramidal syndrome with hypokinesia, mainly axial hypertonia and rest tremor [2,6,9]. The facial expression of the "Wilsonian" patient is often very suggestive: the face is set, the mouth is open, and the hypertonicity of the perioral muscles gives the appearance of a permanent sardonic smile [6,9]. In most cases, symptoms develop very gradually, but a sudden onset after a triggering factor such as trauma or especially surgery under general anesthesia has been described [6]. It should also be noted that WD may be manifested by torticollis [10]. Psychiatric disorders are inaugural in 15% of cases or accompany neurological manifestations [11].

The usual ophthalmological feature of WD is the green pericorneal Kayser-Fleischer ring, which reflects copper deposits in Descemet's membrane. It is carefully examined with a slit lamp and is almost pathognomonic of the disease. However, it can also be observed in cases of severe cholestasis, notably in primary or cryptogenic biliary cirrhosis and in primary sclerosing cholangitis [12]. It is almost always present in patients with neurological disorders [2]. However, in hepatic forms, it may be absent in 25% of cases. If it is absent, the diagnosis of WD cannot be ruled out. In our series, it was present in 42% of cases, three of which were unaccompanied by neurological involvement. The ring disappears in a few years under chelation therapy, and its reappearance in a patient undergoing medical treatment should raise suspicions of poor compliance with treatment [12]. Another ophthalmological manifestation is the much rarer sunflower cataract, visible only on slit-lamp examination [12,13]. These two manifestations do not alter vision [12,14].

Other organ damage may occur in WD. Skin involvement may include hyperpigmentation, azure lunules on the nails, and xerosis of the skin [8,13,15]. Osteoarticular damage, which occurs later, is multifactorial and can affect all joints [9,16]. Endocrine and cardiac manifestations have also been described in WD [3,8,9,13,15,16].

Acute or subacute Coombs-negative hemolytic anemia may be the first sign of WD [3,17]. Most cases have cirrhosis with a low-noise, chronic hemolytic anemia, with a few acute episodes that may precede the hepatic or neurological symptomatology by several years [18]. Thrombocytopenia is often found in Wilsonian patients with prominent splenomegaly [14]. It is secondary both to the hypersplenism caused by cirrhosis and to the direct toxicity of copper, which affects megakaryogenesis by causing platelet production abnormalities [13]. Leukopenia secondary to hypersplenism has also been described in WD [13]. In our series, it was found in four patients.

Renal involvement is almost always present but is usually clinically unobtrusive [19]. Its manifestations are most often related to proximal tubular lesions, much more rarely to glomerular damage [13]. Glomerulonephritis with immune complex or IgA nephrotic syndrome has also been described during the disease itself or under treatment with D-penicillamine [13]. In all cases, they are secondary to copper toxicity [20]. In our series, we found three cases of renal involvement at the time of the diagnosis of Wilson's disease.

Asymptomatic forms are usually detected, ideally around the age of 3 or 4, in the siblings of a child newly diagnosed with WD. Biological signs can be summed up as a moderate and persistent rise in transaminases, but this may be absent even though the copper balance has been positive for many years. Molecular biology can make an important diagnostic contribution here when sibling studies have proved informative.

Biological disturbances specific to WD are based on analysis of the copper balance, which must include ceruloplasminemia, total cupremia, exchangeable copper, enabling calculation of the exchangeable copper/total copper ratio, and 24-hour cupruria. Studies have suggested that the sensitivity of a low ceruloplasmin level for WD ranges from 80% to 99%, although the specificity is lower. Furthermore, in patients with hepatic presentations, a low ceruloplasmin level may be less predictive of WD [3]. However, in 5-10% of patients, this level may approach normal or even be normal. The ceruloplasmin level may, therefore, be a factor in the suspicion, but not the diagnosis, of WD.

Serum copper is generally very low. Total cupremia is made up of ceruloplasmin-bound copper (92%) and ionic-free copper. Total cupremia is normally low but not collapsed, as there is an increase in the free copper fraction. This dosage is often highly variable during the course of the disease and is, therefore, of little practical use [2,3].

Urinary copper levels are essential for diagnosing WD. In symptomatic patients, the level is systematically high (>100 μg/24 h). This level must be confirmed on 24-hour urine samples repeated once or twice, as there is day-to-day variability, influenced in particular by diet. There is only one other situation in which urinary copper levels may be elevated: obstructive liver disease, such as primary biliary cirrhosis or cholestasis. In this situation, both hepatic and urinary copper levels may be elevated, and even the Kayser-Fleischer ring may be present. A urinary copper level <50 μg/24 h in a symptomatic patient in the absence of renal insufficiency, as it cannot be interpreted in the case of renal impairment, virtually rules out the diagnosis of WD. If the urinary copper value is between 50 and 100 μg/24 h, further investigations are warranted. This assay is also extremely important for monitoring treatment efficacy and compliance [21].

Exchangeable copper is a new assay for free copper, and the exchangeable copper/total copper ratio, or REC, is certainly the best method of analysis. It is an excellent diagnostic biomarker, with a sensitivity close to 100% when its value is greater than 18.5%. The REC ratio can be used to differentiate Wilsonian hepatopathy from hepatopathy of other etiologies (non-alcoholic steatohepatitis [Nash], autoimmune, or infectious, whether in children or adults). In family screening for WD, the REC ratio can also be used to differentiate heterozygous carriers or healthy subjects from diseased subjects. The determination of exchangeable copper provides a direct and accurate measure of copper overload. At the time of diagnosis, it provides information on the spread and severity of the disease [1,2].

In neurological forms, cerebral CT scans may be normal, even when the Kayser-Fleischer ring is present. In most cases, it shows a characteristic bilateral hypodensity of the lenticular nuclei. MRI, on the other hand, is almost always pathological in neurological forms, revealing gray-nucleus hypersignals of variable size and shape on T2-weighted and FLAIR sequences involving the putamen, striatum, and pallidum, as well as cortical atrophy and white matter changes [12,22]. Hyper-signaling in the midbrain around the red nucleus and substantia nigra can give the appearance of a "panda sign," which is most often seen in Wilsonian patients and has been shown to be fairly specific for WD but not very sensitive [3,22]. Brain imaging is also useful for monitoring the evolution of lesions under treatment.

WD is one of the few treatable metabolic diseases. Treatment must be initiated early and for life, because once irreversible damage has set in, the effect of treatment is limited, and the patient's life quality is permanently compromised. Treatment involves a diet that avoids copper-rich foods (mushrooms, liver, seafood, nuts, and chocolate). Copper chelators are the mainstay of treatment (DPenicillamine [DP] or Trolovol®, and Triethy-tetramine (TETA) or Trientine®). Their doses are administered two to four times a day, without food, i.e., one hour before or two hours after meals. They are increased to an approximate dose based on body weight of around 20 mg/kg/d for initial treatment. Inhibitors of digestive copper absorption (mainly zinc acetate or sulfate) represent another possible therapeutic approach [20]. Treatment must be monitored both clinically and biologically (cupruria). The effectiveness of therapeutic management is only observed several months after its introduction, notably through normalization of the liver balance. Liver transplantation may be considered in cases of fulminant hepatitis [6]. In presymptomatic or paucisymptomatic forms, it is generally accepted to treat with zinc, which is inexpensive and has few side effects [23]. The difficulty in our country is to obtain D-penicillamine, as this product is not available in Morocco.

The deaths recorded in our series could be explained either by a delay in diagnosis of this condition, given the difficulty of biological confirmation of the disease (the high cost of the copper test), or by practitioners' lack of knowledge of the disease, or by a delay in starting treatment or poor compliance. All of this adds up to diagnostic difficulties in our low-income country, especially as the copper test is required to screen all siblings as part of a family survey.

These days, two genetic study strategies are used: indirect family diagnosis by haplotype analysis or direct diagnosis by mutation search [17,18].

Our series data align closely with previous Moroccan studies [24-26], as demonstrated in Table 3. Interestingly, we observe a notable prevalence of neurological manifestations in series from Western nations [27,28] when compared to their Moroccan counterparts.

**Table 3:** Comparison of our data with other literature series.

		Idrissi et al. (Fès, Morocco) [24]	Ouharakat et al. (Rabat, Morocco) [25]	Abbasi et al. (Marrakech, Morocco) [26]	Manolaki et al. (Athens, Greece) [27]	Dhawan et al. (London, UK) [28]	Our series (Oujda, Morocco)
Number of cases		20	10	46	57	74	24
Length of study (years)		7	12	11	21	37	9
Consanguineous marriage (%)		65%	60%	67.4%	-	-	54%
Liver manifestations (%)		85%	40%	-	33%	77%	75%
Neurological manifestations (%)		15%	30%	-	-	70%	25%
Asymptomatic		15%	20%	8.6%	31%	23%	21%
Low serum ceruloplasmin (<0.2 g/l)		85%	90%	93.3%	72%	77%	88%
24 h urinary copper >100 μg/day		89.4%	90%	97%	56%	95%	100%
Kayser-Fleischer ring (%)		70%	40%	61%	38%	46%	54%
Treatment	D-penicillamine	85%	100%	93%	95%	42%	75%
	Zinc acetate/sulfate	15%		4.3%	1.7%	1.3%	25%
	Combination of penicillamine and zinc					48%	
	Trientine	0	0	0	16%	9.5%	0
Evolution	Good	60%	80%	61%	96%		63%
	Death	20%	20%	38%	3.5%	25%	21%
	Lost sight	20%	0%	4%			16%

Limitations of the study

Our study was limited by the fact that we live in a low-income country, which meant that we were unable to carry out a complete copper workup on all patients, in particular the REC ratio. On the other hand, we were unable to follow the progress of all our patients, as four were lost to follow-up.

## Conclusions

Our study enabled us to identify some specificities in our country, including the underestimated frequency, knowing that our region is very rich in consanguinity. There is also the diagnostic delay and the marked predominance of hepatic manifestations compared to neurological manifestations, which are much less pronounced. This observation corroborates other Moroccan series and contradicts series from other countries where a significant percentage of neurological involvement is found. Following our results, which identified a 12-year-old girl with a purely neuropsychiatric presentation, raising awareness about the neurological symptoms of Wilson's disease is crucial, as neurological disorders can present with silent or even absent hepatic involvement.
